# A Systematic Review on the Role of Antibiotics and Analgesics in Systemically Ill Patients Undergoing Tooth Extraction

**DOI:** 10.7759/cureus.59711

**Published:** 2024-05-06

**Authors:** Akshaya Subhashinee Dhanasekaran, Muthalagappan P L, ShriKrishna Prasanth, Ezhil Dharshini A, Koushika Mohan, Ananthanarayanan V

**Affiliations:** 1 Oral and Maxillofacial Surgery, Sri Ramaswamy Memorial (SRM) Dental College, Ramapuram Campus, Chennai, IND

**Keywords:** infection, systemically ill, extraction, analgesics, antibiotics

## Abstract

Antibiotics are commonly prescribed as a preventive measure, particularly post-tooth extraction, aiming to minimize the risk of infection. Preemptive analgesia functions by disrupting the nervous system's ability to encode pain stimuli, thus preventing the formation of pain memory. Dentists often recommend analgesics and antibiotics either as adjuncts or sole treatments for various dental conditions, offering both efficacy and cost-effectiveness.

A comprehensive literature search was conducted across multiple databases, including PubMed, Cochrane Central Register of Controlled Trials (CENTRAL), Science Direct, and Lilac, using MeSH terms relevant to the role of antibiotics and analgesics in systemically ill patients undergoing tooth extraction. Out of 178 articles screened, 83 underwent full-text assessment for eligibility, and six were selected for qualitative analysis. The review process adhered to the Preferred Reporting Items for Systematic Reviews and Meta-Analyses (PRISMA) guidelines, ensuring methodological rigor and transparent reporting. Across diverse study populations, the role of antibiotics and analgesics consistently demonstrated a statistically significant impact. Hence, the utilization of analgesics and antibiotics plays a pivotal role in preventing infection following tooth extraction in systemically ill patients, thereby promoting optimal oral hygiene and overall health.

## Introduction and background

Antibiotic prophylaxis is beneficial in preventing problems in high-risk patients, and it is also recommended in situations of active infection during surgery. Historically, dentists have employed preventive antibiotics to prevent Dry sockets and Surgical site infection. Amoxicillin, either alone or in combination with clavulanic acid, is the most often used antibiotic for preventing postoperative infection following L3M extraction. Clindamycin, doxycycline, erythromycin, and metronidazole are some of the other antibiotics [1]. Dentists prescribe about 10% of all antibiotics in primary care. Antibiotics are typically recommended as a preventative measure, especially following tooth extraction [2]. 

Antibiotics are occasionally used post-extraction in clinical practice to avoid these problems. It is a contentious practice, though. After extraction, the prescription of antibiotics affirms the view held by some that antibiotics are necessary for the extraction wounds to heal unevenly. A minority of people maintain a different perspective, contending that even in cases of surgical tooth extraction, the possibility of developing infectious problems after the procedure is insufficient to justify the use of antibiotics. This second group goes on to say that the body's defense mechanisms allow healing to occur naturally without the need for antibiotics [3].

Analgesia administered ahead of the painful stimulus is known as pre-emptive analgesia. The goal of providing analgesia before the unpleasant stimuli is to avoid or lessen subsequent pain. Pre-emptive analgesia works by inhibiting or preventing the nervous system from developing any "memory" of the pain stimuli. The potential to enhance postoperative pain management is of clinical interest. Numerous surgical treatments have been proven to benefit from it, including tonsillectomy, inguinal hernia repair, cholecystectomy, orthopedic surgeries, and extraction of molar teeth.

The effectiveness of tramadol as an analgesic during various dental operations has been described. Based on clinical research, tramadol may possess characteristics similar to those of a local anesthetic. However, the preemptive analgesic impact of tramadol has not been thoroughly examined in many research. Comparing the effectiveness of tramadol administered prior to or right after surgical extraction of an impacted mandibular third tooth under local anesthetic was the aim of this investigation [4].

Medication-related osteonecrosis of the jaw (MRONJ) is characterized by the exposure of bone or a bone that can be probed through either an intraoral or extraoral fistula in the maxillofacial region. It is defined by the failure of the exposed bone to heal within an eight-week period, particularly in patients with a medical history indicating the use of a bone-modifying agent (BMA) and no prior exposure to radiation in the head and neck. First identified in 2003, bisphosphonates emerged as the inaugural bone-modifying agent linked to the development of MRONJ. In cases where conservative treatment has failed or when teeth extraction cannot be postponed any longer, systemic antibiotics are frequently administered to patients undergoing surgery, whether they are intravenous or oral bisphosphonates [5].

However, recent meta-analyses and systematic reviews have concluded that amoxicillin alone does not effectively prevent post-operative infections following third molar extractions. Moreover, a separate review examining antibiotic use in tooth extractions revealed a range of clinical complications, including dry socket, pain, fever, swelling, and adverse reactions such as nausea and diarrhea. Additionally, the use of antibiotics in the oral cavity raises concerns beyond immediate clinical outcomes, including potential impacts on the oral microbiome. Consequently, there is a critical need to assess the long-term effects of amoxicillin administration for third molar extractions on both the oral microbiome and the persistence of infections [6].

N-acetyl-aminophenol, the chemical formula for acetaminophen or paracetamol, is an analgesic and an antipyretic. Acetaminophen has been marketed as a pain and fever reliever "over the counter" due to its safer clinical profile within the authorized dosage. When other nonsteroidal anti-inflammatory medicines are not recommended for a patient, paracetamol is the preferred painkiller [7].

While the importance to specific species in this condition is significantly higher than in the overall incidence of bacteremia, the pathogenic role of the anaerobic periodontal pathogens group in causing infective endocarditis remains an entirely separate question. It has long been thought that streptococci should be the primary focus in the fight against infective endocarditis; in risk patients, this condition typically manifests itself within two weeks after a dental operation. In addition to eliminating bacteria, antibiotic prophylaxis also prevents bacteria from adhering to surfaces [8].

Furthermore, adding betamethasone to the flurbiprofen and acetaminophen injections may offer the best possible pain and swelling control when extracting impacted wisdom teeth under general anaesthesia. Furthermore, the purpose of administering analgesics under general anaesthesia is to offer proactive analgesia, which aims to efficiently manage pain following the extraction of impacted teeth. In particular, when administered for pre-emptive analgesia, acetaminophen injection is also recognized to be beneficial in managing postoperative pain. Therefore, when third molars are extracted under general anaesthesia, injections of flurbiprofen, acetaminophen, and betamethasone provide effective pain and symptom management with few side effects [9].

## Review

This study aimed to evaluate the effectiveness of antibiotics and analgesics in patients with systemic illness undergoing tooth extraction.

Materials and methods

Eligibility criteria were set for a systematic review of randomized controlled trials. Included were original articles with full-text availability, focusing on the role of antibiotics and analgesics in systemically ill patients undergoing tooth extraction. Excluded were animal studies and articles lacking full-text access [10].

**Figure 1:**
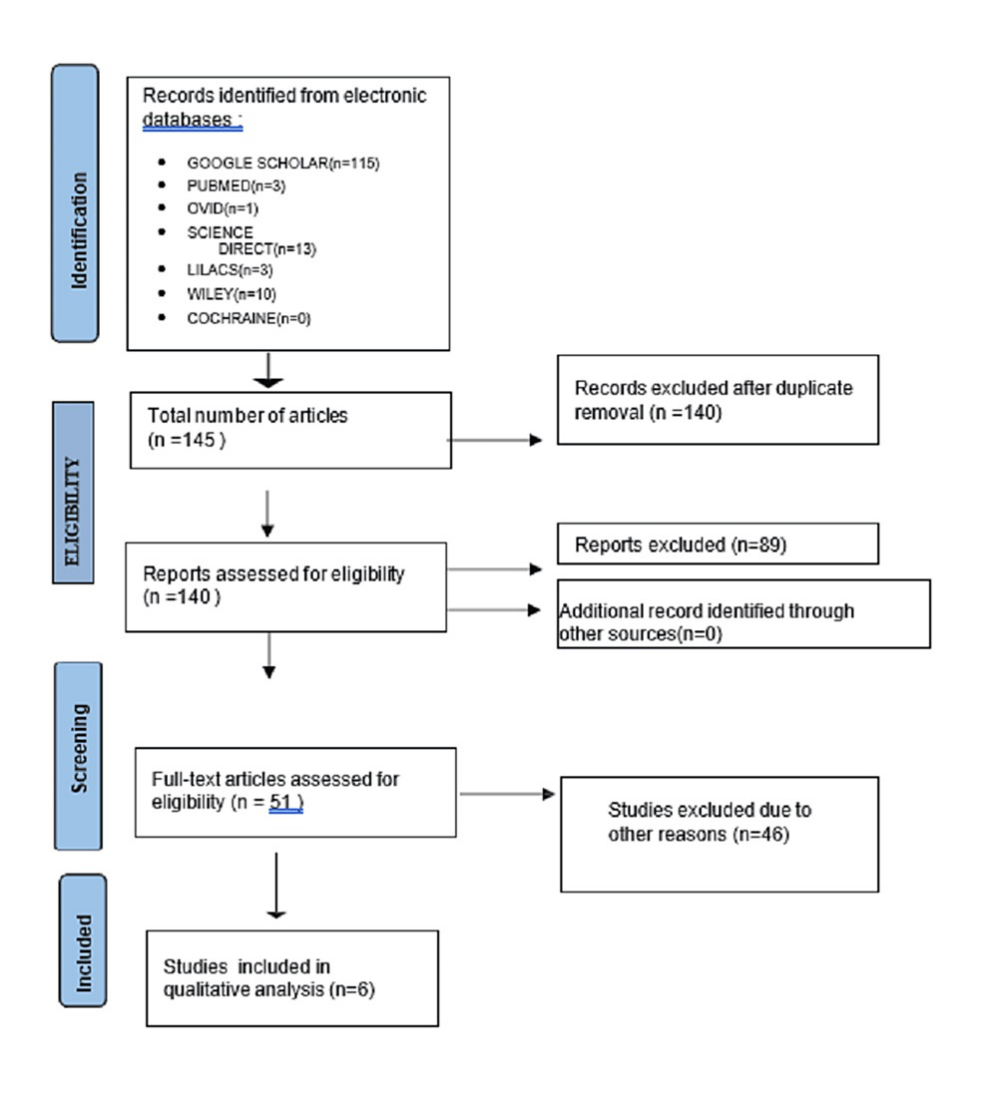
Flow diagram showing the number of studies identified, screened, and assessed for eligibility, excluded and included in the systematic review

A comprehensive search strategy was employed across various databases to gather relevant literature regarding the role of antibiotics and analgesics in systemically ill patients undergoing extraction. Original articles and research papers were sought on platforms such as PubMed, Google Scholar, Science Direct, Lilacs, Wiley, and Cochrane. The search utilized MeSH terms including "ANTIBIOTICS," "ANALGESICS," "EXTRACTION," and "SYSTEMICALLY ILL PATIENT" to ensure comprehensive coverage. Adhering to the Preferred Reporting Items for Systematic Reviews and Meta-Analyses (PRISMA) guidelines, modifications to the MeSH terms were made for each search engine to optimize the retrieval of pertinent studies (Figure 1).

**Table 1:** Characteristics of interventions in included studies

Author names	Year	Sample size	Patient characteristics	Number of patients indicated for receiving medication prophylaxis	Type of drugs
Pergolizzi et al. [10]	2020	183	Patients with post-surgical pain (3^rd^ molar extraction)	120	Codeine, Hydrocodone, Hydromorphone, Morphine, Oxycodone, Tapentadol
Al-Bazie et al. [11]	2016	2232	The study included individuals with a prior diagnosis of head and neck cancer who had undergone radiotherapy and were recommended for tooth extraction	89	Amoxicillin, Beta-lactam antibiotics, Clindamycin
Sato et al. [12]	2021	6,62,435	Patients 18 years of age and older	5,47,528	Amoxicillin, Cephalosporin, Broad spectrum penicillin, Macrolide, Quinolone.
Karthikeyan et al. [13]	2020	7889	Patients of age group up to 60 years	3613	Paracetamol, Aceclofenac, Diclofenac, Kerorolac, Tramadalol, Aceclofenac + Paracemol combination analgesic and Paracetamol + Diclofenac drug of combination
Gopalasamy et al. [14]	2021	4471	Patients 40 years and above undergoing extraction	4455	Amoxicillin, Metronidazole, Clavulanic acid, Novomox, Cefixime
Ahmad et al. [15]	1997	5171	Patients undergoing third molar extraction	890	Acetaminophen, codeine, zomepirac, flurbiprofen, ibuprofen, aspirin, acetaminophen (650)-codeine (60)

**Table 2:** Presents the outcome data gathered from the studies included in the analysis

Author name	Year	Effect measure	Results
Pergolizzi et al. [10]	2020	183	In a controlled trial involving 30 patients undergoing dental surgery, the effectiveness of oxaprozin 1200 mg was compared to Naproxen 550 mg alongside a placebo. Trismus, a condition causing difficulty in mouth opening, was assessed before and after dental extraction, while pain levels were measured using a visual analog scale. A combination of ibuprofen and oxycodone (400/5 mg) showed superior pain relief compared to oxycodone alone. However, this combination did not demonstrate statistically significant superiority over ibuprofen alone at a dose of 400 mg. Additionally, a study involving a meloxicam transdermal patch in 55 patients with Dental Impaction Pain Management (DIPM) reported decreased pain scores within 30 minutes of wearing the patch.
Al-Bazie et al. [11]	2016	2232	Among a cohort of 2232 patients, 225 had a history of radiotherapy to the maxillofacial region. Among these, 225 patients were referred to or self-reported for dental extractions. Additionally, 89 patients presented with nonrestorable teeth within the radiation exposure area in the maxilla, mandible, or both. Three patients (3.4%) experienced delayed healing, which was successfully managed conservatively and resolved within three months.
Sato et al. [12]	2021	6,62,435	Following the application of exclusion criteria, a total of 662,435 eligible dental visits were identified. Of these, 547,528 visits (83%) were prescribed antibiotics, with 366,639 visits (55%) receiving third-generation cephalosporins. Patient characteristics remained consistent across the years, showing no significant changes.
Karthikeyan et al. [13]	2020	7889	Paracetamol emerged as the top choice for analgesia among different age groups, with 896 individuals aged 0-20 years, 2356 individuals aged 21-60 years, and 388 individuals aged 60 years and above preferring it. Following closely, Aceclofenac ranked as the second most favored analgesic, selected by 157 individuals aged 0-20 years, 1234 individuals aged 21-60 years, and 259 individuals aged 60 years and above.
Gopalasamy et al. [14]	2021	4471	Among these patients, approximately 3890 individuals were prescribed amoxicillin post-extraction, while the next two most commonly prescribed antibiotics were metronidazole, with 303 patients receiving it. Another combination, amoxicillin with clavulanic acid, was prescribed to about 120 patients, and amoxicillin with metronidazole was administered to 115 patients. Notably, in the age group of 42-65 years, amoxicillin and beta-lactam inhibitors were frequently utilized.
Ahmad et al. [15]	1997	5171	Better pain relief was obtained from Acet-Cod-60 compared with 600 and 1000 mg of acetaminophen. Ketorolac at doses of 10 or 20 mg was more efficient than Acet-Cod-60, with statistically significant differences for the PPAR and TOTPAR scores, but not for the others.

**Table 3:** Shows the bias assessment of the included studies + = Low risk of bias; - = High risk of bias; ? = unclear risk of bias

Author name, year	Random sequence generation	Allocation concealment	Blinding of outcome	Incomplete outcome data	Blinding of participants and personnel	Selective reporting	Judgmental Bias
Pergolizziet al [10]	+	-	+	+	?	-	+
Al- Bazie et al. [11]	+	-	+	+	-	-	+
Sato et al. [12]	+	+	+	+	?	+	+
Karthikeyan et al. [13]	+	+	+	+	+	+	+
Gopalasamy et al. [14]	+	+	?	?	-	+	?
Ahmad et al. [15]	+	+	-	+	+	-	+

Despite widespread agreement that patients at risk of osteoradionecrosis (ORN) should take peri-extraction antibiotics, there is a noticeable dearth of specific information regarding the kind, dosage, and timing of antibiotic prophylaxis. Given the wide range of responses, it is clear that there is insufficient evidence to provide clear rules. When comparing patients who underwent surgery alone with those who had prior radiation, it was evident that the former group benefited more from pre-extraction antibiotics. Before considering augmentin or a combination of amoxicillin and metronidazole, the non-allergic patient was recommended to take prophylactic amoxicillin. The use of clindamycin, metronidazole, and cephalosporins in combination has been recommended for patients allergic to penicillin. despite the fact that amoxicillin, metronidazole, and clindamycin are options that align with recent recommendations. The duration of the course ranged from three to 28 days and, in one case, continued until the patient had fully recovered [10-15].

The lack of a definitive oral health protocol for people at risk of ORN is revealed by this activity. Overprescribing is also clearly seen in patients who are at risk of ORN. The emergence of antibiotic resistance is a direct result of the overuse of these drugs. Furthermore, a lethal adverse host reaction might occur when an antibiotic drug is administered in a high dosage [16].

It is interesting to note that, despite the fact that the patient's subjective assessments of their pain and the objective measurements of swelling clearly indicated that the ketoprofen regimen was superior to the acetaminophen regimen, there was no statistically significant difference between the treatments in terms of preference scores and global evaluation. When it came to mouth opening, a clinically significant indicator of inflammation known as "reduced function," there was no discernible statistical difference between the therapies. The outcomes of measuring mouth openness and evaluating swelling and pain differ from each other [17].

With its low toxicity, widespread availability, affordability, and strong ability to compete with food absorption, amoxicillin is one of the most popular antibiotics. Clindamycin is another antibiotic that is commonly prescribed by dentists about 24.3% of the time. Patients with allergies to beta-lactam antibiotics have an alternate antibiotic choice, clindamycin, which influences the usage.

On the other hand, 81.4% of dentists in this survey felt that patients shouldn't be given antibiotics following a routine tooth extraction. Reasonable drug criteria must be met before antibiotics are prescribed, including accurate patient assessment, diagnosis, and medication selection. Antibiotic resistance can be accelerated by the unchecked use of broad-spectrum antibiotics. Consequently, while prescribing antibiotics to patients, dentists should use caution. Before administering antibiotics to a patient following tooth extraction, it is important to take into account various elements, including the patient's oral hygiene, the severity of the process, and the range of tooth extraction methods [18].

The issue of antibiotic overprescribing is complex. Healthcare providers have been offered a number of strategies. Possible interventions include media campaigns, audits and feedback, educational seminars and lectures, delayed prescribing, clinical decision support systems, and the dissemination of guidelines to providers. There is debate concerning practice recommendations. The greatest obstacle to the effective application of clinical practice guidelines is the concern that practitioners will lose some of their independence as professionals as a result of following the guidelines, even though guidelines are perceived as useful for continuing education and daily clinical decision-making [19]. In order to prevent infections caused by an unsterile environment, a lengthy or difficult procedure, patients with pre-existing immunosuppression, metabolic disorders (such as diabetes), underlying cardiac conditions, or pre-existing dental conditions, a small percentage of respondents explained that they administered post-extraction Antibiotic. There were 56 responders (38.6% of the total) who did not know whether using antibiotics would lessen the likelihood of infection after M3 extractions [20] (Tables 1-3).

This study systematically evaluated the role of antibiotics and analgesics in preventing post-tooth extraction infections among systemically ill patients. Through a rigorous literature search and adherence to PRISMA guidelines, five studies were identified for qualitative analysis out of 178 screened articles. The findings consistently indicated a statistically significant impact of these interventions in disrupting pain stimuli encoding, thus averting the formation of pain memory. Dentists' recommendations of antibiotics and analgesics, either as adjuncts or sole treatments, underscore their recognized efficacy and cost-effectiveness in dental practice.

Moving forward, this research emphasizes the critical importance of preemptive analgesia in mitigating pain and infection risk following tooth extraction, particularly in patients with systemic illnesses. While the reviewed studies provide valuable insights, further investigation is warranted to optimize dosing, duration, and selection of antibiotics and analgesics. Future research directions should include comparative effectiveness studies and the exploration of alternative non-pharmacological pain management strategies. Addressing these gaps will enhance patient care and contribute to the judicious use of antibiotics in dental practice.

## Conclusions

In conclusion, despite widespread recognition of the need for peri-extraction antibiotics in patients at risk of ORN, there remains a lack of consensus on specific protocols, leading to overprescription and concerns about antibiotic resistance. While certain antibiotics like amoxicillin and clindamycin are commonly recommended, varying durations and dosages highlight the need for standardized guidelines. Additionally, the efficacy of pain management medications like ketoprofen versus acetaminophen underscores the complexity of treatment evaluation. Dentists must exercise caution in antibiotics prescribing, considering factors such as patient oral hygiene and procedural severity to mitigate risks of overuse and resistance development.
